# The initial hormone receptor/HER2 subtype is the main determinator of subtype discordance in advanced breast cancer: a study of the SONABRE registry

**DOI:** 10.1007/s10549-021-06472-5

**Published:** 2022-01-13

**Authors:** Marissa Meegdes, Khava I. E. Ibragimova, Dorien J. A. Lobbezoo, Ingeborg J. H. Vriens, Loes F. S. Kooreman, Frans L. G. Erdkamp, M. Wouter Dercksen, Birgit E. P. J. Vriens, Kirsten N. A. Aaldering, Manon J. A. E. Pepels, Linda M. H. van de Winkel, Jolien Tol, Joan B. Heijns, Agnes J. van de Wouw, Natascha A. J. B. Peters, Ananda Hochstenbach-Waelen, Marjolein L. Smidt, Sandra M. E. Geurts, Vivianne C. G. Tjan-Heijnen

**Affiliations:** 1 Department of Internal Medicine, Division of Medical Oncology, Maastricht University Medical Centre, Maastricht, The Netherlands; 2 GROW School for Oncology and Developmental Biology, Maastricht University, Maastricht, The Netherlands; 3 Department of Pathology, Maastricht University Medical Centre, Maastricht, The Netherlands; 4 Department of Internal Medicine, Zuyderland Medical Centre, Sittard-Geleen, The Netherlands; 5 Department of Internal Medicine, Máxima Medical Centre, Veldhoven, The Netherlands; 6 Department of Internal Medicine, Catharina Hospital, Eindhoven, The Netherlands; 7 Department of Internal Medicine, Laurentius Hospital, Roermond, The Netherlands; 8 Department of Internal Medicine, Elkerliek Hospital, Helmond, The Netherlands; 9 Department of Internal Medicine, St Anna Hospital, Geldrop, The Netherlands; 10 Department of Internal Medicine, Jeroen Bosch Ziekenhuis, Den Bosch, The Netherlands; 11 Department of Internal Medicine, Amphia Hospital, Breda, The Netherlands; 12 Department of Internal Medicine, Viecuri Medical Centre, Venlo, The Netherlands; 13 Department of Internal Medicine, St. Jans Gasthuis, Weert, The Netherlands; 14 Department of Surgery, Maastricht University Medical Centre, Maastricht, The Netherlands

**Keywords:** Breast cancer, Metastatic disease, Hormone receptor, HER2 receptor, Subtype, Biopsy

## Abstract

**Purpose:**

The hormone receptor (HR) and human epidermal growth factor receptor 2 (HER2) are the main parameters in guiding systemic treatment choices in breast cancer, but can change during the disease course. This study aims to evaluate the biopsy rate and receptor subtype discordance rate in patients diagnosed with advanced breast cancer (ABC).

**Methods:**

Patients diagnosed with ABC in seven hospitals in 2007–2018 were selected from the SOutheast Netherlands Advanced BREast cancer (SONABRE) registry. Multivariable logistic regression analyses were performed to identify factors influencing biopsy and discordance rates.

**Results:**

Overall, 60% of 2854 patients had a biopsy of a metastatic site at diagnosis. One of the factors associated with a reduced biopsy rate was the HR + /HER2 + primary tumor subtype (versus HR + /HER2- subtype: OR = 0.68; 95% CI: 0.51–0.90). Among the 748 patients with a biopsy of the primary tumor and a metastatic site, the overall receptor discordance rate was 18%. This was the highest for the HR + /HER2 + primary tumor subtype, with 55%. In 624 patients with metachronous metastases, the HR + /HER2 + subtype remained the only predictor significantly related to a higher discordance rate, irrespective of prior (neo-)adjuvant therapies (OR = 7.49; 95% CI: 3.69–15.20).

**Conclusion:**

The HR + /HER2 + subtype has the highest discordance rate, but the lowest biopsy rate of all four receptor subtypes. Prior systemic therapy was not independently related to subtype discordance. This study highlights the importance of obtaining a biopsy of metastatic disease, especially in the HR + /HER2 + subtype to determine the most optimal treatment strategy.

**Supplementary Information:**

The online version contains supplementary material available at 10.1007/s10549-021-06472-5.

## Introduction

The systemic therapy choice in patients diagnosed with advanced—metastatic—breast cancer (ABC) is primarily based on the receptor subtype. The receptor subtypes are derived from the hormone receptor (HR) status and human epidermal growth factor receptor 2 (HER2) status, specified as HR + /HER2-, HR + /HER2 + , HR-/HER2 + , and triple negative (TN). In recent years various treatment options became available for ABC. These options have included mTOR, PI3K, and CDK4/6 inhibitors for patients with HR + /HER2- disease [1–3], HER2-targeted therapies for HER2 + disease [4–8], PARP inhibitors for patients with BRCA1/2 mutated, HER2-negative disease [9], and checkpoint inhibitors for PD-L1-positive TN disease [10]. Following these developments, it has become increasingly important to determine the receptor subtype of the tumor in guiding systemic treatment choices.

The receptor subtype of a recurrent tumor site can, however, be different from the receptor subtype of the primary tumor, referred to as subtype discordance. Several studies have reported varying discordance rates for the estrogen receptor (ER), progesterone receptor (PR), and HER2 status between primary breast tumor and loco-regional or distant recurrences. A recent meta-analysis, including 39 prospective and retrospective studies, found discordance rates between the primary tumor and the metastatic site of 19% for ER, 31% for PR, and 10% for HER2, respectively [11]. This growing knowledge about high receptor discordance rates has resulted in a worldwide consensus on the importance of obtaining tissue of a metastatic site [12]. In the Netherlands, this led to an explicit recommendation in 2012 that, whenever possible, the initial metastatic site needs to be biopsied for receptor assessment apart from confirming presence of metastatic disease [13]. In terms of its clinical implication, a change in receptor subtype often leads to an adjustment of the treatment strategy. A pooled analysis of the prospective BRITS and DESTINY trials, including 289 patients with suspected breast cancer recurrence (loco-regional recurrence and distant recurrence), revealed that the systemic treatment choice was adjusted in 14% of patients when biopsy results of the recurrent disease became available [14].

Important limitations of prior studies reporting on subtype discordance are the focus on specific metastatic sites or both loco-regional recurrences and metastatic sites in their study sample [15–22] and a small sample size for regression and subgroup analysis [23–25]. Considering these issues, we present a large real-world study, including all patients diagnosed with ABC, irrespective of metastatic sites and systemic treatment given. We aimed to assess the biopsy rate and the factors associated with taking a biopsy of a metastatic site at ABC diagnosis. Next, we aimed to determine the receptor subtype discordance rate between the primary tumor and a metastasis. Finally, we aimed to evaluate first-line systemic treatment choices in relation to the occurrence of subtype discordance.

## Methods

### Southeast Netherlands advanced BREast cancer (SONABRE) registry

Data for this study were obtained from the SONABRE Registry (NCT-03577197), an observational cohort study aiming to include all patients aged 18 years and above, with de novo or recurrent ABC in the Southeast of the Netherlands. Specially trained registrars retrospectively collected data from medical files, including patient and tumor characteristics and treatment information (i.e., local and systemic therapy) for both primary tumor and metastatic disease. The Medical Research Ethics Committee of Maastricht University Medical Center + approved the registry (15-4-239).

### Patient selection

For this study, we selected all patients diagnosed with ABC in 2007–2018 from seven hospitals, including one academic, four teaching, and two non-teaching hospitals. All patients were eligible for assessing the biopsy rate at time of ABC diagnosis. For assessing receptor and subtype discordance rates, only patients with a known subtype of both the primary tumor and a metastatic site at time of ABC diagnosis were eligible.

### Definitions

Resection material, or if unavailable biopsy tissue, of the primary tumor was used to determine the subtype of the primary tumor. HER2 positivity was defined as a positive in situ hybridization (ISH) result or immunohistochemistry (IHC) score of 3 +. If the HER2 status was not reported, but HER2-targeted therapy was given in the (neo-)adjuvant setting, HER2 status was considered positive. ER and PR positivity were defined as positive nuclear staining of ≥ 10%. HR status was considered positive in case of ER and/or PR positivity and ER result was leading in the absence of PR status. When ER status of the primary tumor was unknown and ABC diagnosis was since the year 2002, ER receptor was considered positive when endocrine therapy was given in (neo-)adjuvant setting. Metastatic-free interval (MFI) was defined as the interval between date of primary tumor diagnosis and date of ABC diagnosis.

### Endpoints and statistical analyses

The first study goal was to determine the proportion of patients with a biopsy assessment of a metastatic site at ABC diagnosis. A multivariable logistic regression analysis was used to examine the following pre-specified factors possibly associated with a biopsy assessment: period of ABC diagnosis, age at ABC diagnosis, subtype of primary tumor, metastatic site, number of primary tumors, and metastatic-free interval (MFI).

The second study goal was to define subtype concordance and discordance rates for patients with complete information on the receptor subtype of both the primary tumor and a metastatic site at time of ABC diagnosis. Patients with two or more primary tumors with different subtypes were excluded from this analysis. A univariable logistic regression analysis was performed to find associated predictors for discordance based on prior research, including age, WHO performance status, genetic mutation, histology and subtype of the primary tumor, and MFI [21, 24–26]. Additionally, a separate analysis was performed including the type of adjuvant therapies while excluding patients with de novo ABC. All potential predictors with a *P* value of < 0.2 in the univariable analyses were included in the main multivariable model. All reported *P* values are two-sided and considered borderline significant at a value of ≤ 0.10 and statistically significant at ≤ 0.05.

Lastly, we selected the primary receptor subtype where discordance was most prevalent and described the initial systemic treatment choices per metastatic receptor subtype.

## Results

### Patient and tumor characteristics

Among the 2854 patients included, 75% were younger than 75 years and 79% had a good performance status (WHO 0–1) at diagnosis of ABC (Table 1). The large majority (84%) had a primary breast cancer diagnosis since the year 2000 and 65% had a HR + /HER2- primary tumor subtype. Twenty-three percent of patients had de novo ABC and 42% a MFI over 60 months and 27% had bone-only metastatic disease and 11% only visceral metastases. Of patients with metachronous metastases, 76% had received (neo-)adjuvant systemic therapy.

**Table 1:** Baseline characteristics of patients at moment of ABC diagnosis, for the total group and the subgroup of patients with known subtype of the primary tumor and an initial metastatic site

	Total	Known subtype of primary tumor and metastatic lesion
	*N* = 2854	*N* = 748
	*N* (%)	*N* (%)
Gender		
Female	2828 (99)	741 (99)
Age at ABC diagnosis		
Median age (IQR)	65 (55–75)	62 (52–72)
18–55 years	710 (25)	249 (33)
56–75 years	1421 (50)	377 (51)
> 75 years	723 (25)	122 (16)
Comorbidity at ABC diagnosis		
Any	1567 (55)	359 (48)
WHO performance score at ABC diagnosis		
WHO 0–1	1540 (79)	500 (85)
WHO ≥ 2	417 (21)	91 (15)
Unknown	897	157
Period of primary breast cancer diagnosis		
< 1990	84 (3)	4 (1)
1990–1999	388 (13)	22 (3)
2000–2009	1306 (46)	354 (47)
2010–2018	1076 (38)	368 (49)
Period of ABC diagnosis		
2007–2009	715 (25)	97 (13)
2010–2012	663 (23)	139 (19)
2013–2015	747 (26)	251 (33)
2016–2018	729 (26)	261 (35)
Histology primary tumor		
Ductal	2152 (75)	608 (81)
Lobular	511 (18)	117 (16)
Other/unknown	191 (7)	23 (3)
Number of primary tumors		
1	2492 (87)	709 (95)
≥ 2	362 (13)	39 (5)
Subtype primary tumor		
HR + /HER2-	1397 (65)	508 (68)
HR + /HER2 +	276 (13)	91 (12)
HR-/HER2 +	154 (7)	53 (7)
TN	323 (15)	96 (13)
Unknown	704	N.A
HR + /HER2?	488	N.A
HR-/HER2?	45	N.A
HR unknown	171	N.A
Number of metastatic sites		
Single	1281 (45)	296 (40)
Multiple	1573 (55)	452 (60)
Initial metastatic sites		
Bone only	779 (27)	162 (22)
Soft tissue only	113 (4)	35 (5)
Visceral only	326 (11)	91 (12)
CNS only	63 (2)	8 (1)
Multiple sites	1573 (55)	452 (60)
Metastatic-free interval		
< 3 months/de novo	652 (23)	124 (17)
3–24 months	405 (14)	112 (15)
25–59 months	606 (21)	233 (31)
≥ 60 months	1191 (42)	279 (37)
(Neo-)adjuvant treatment^a,b^		
Any systemic therapy	1665 (76)	488 (78)
Endocrine therapy	1282 (58)	378 (61)
HER2-targeted therapy	161 (7)	69 (11)
Chemotherapy	1151 (52)	375 (60)
Radiotherapy on primary tumor	1340 (66)	384 (64)

*ABC* advanced breast cancer, *CNS* central nervous system, *HER2* Human Epidermal growth factor Receptor 2, *HR* hormone receptor, *IQR* interquartile range, *N.A*. not applicable, *TN* triple negative, *WHO* World Health Organization

^a^Sum of percentages exceeds 100 because multiple options are possible

^b^Among patients with recurrent metastases only (excluding patients with de novo ABC)

### Biopsy assessment

**Fig. 1:**
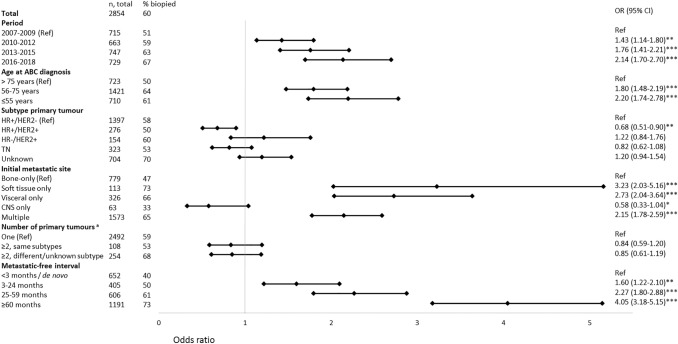
Factors associated with undergoing a biopsy of a metastatic site at time of ABC diagnosis, by multivariable logistic regression analysis. ^a^In case of two or more primary tumors, the subtype of the first primary tumor is reported. *Not statistically significant, but borderline (*P*< 0.10), ** *P*< 0.05, *** *P*< 0.001

Overall, 60% of patients had a biopsy of a metastatic site at presentation. Per type of hospital, biopsy rate was 73% in the academic center, 60% in teaching hospitals, and 50% in non-teaching hospitals. A more recent period of ABC diagnosis was associated with a higher biopsy rate: 67% in 2016–2018 compared with 51% in 2007–2009 (OR = 2.14; 95% CI: 1.70–2.70) (Fig. 1). An independent higher biopsy rate of distant disease was further observed in younger patients (56–75 years versus > 75 years: OR = 1.80; 95% CI: 1.48–2.19; ≤ 55 years versus > 75 years: OR = 2.20; 95% CI: 1.74–2.78), a metastatic site other than bone-only (e.g., for soft tissue only (OR 3.23 = 95% CI: 2.03–5.16) and for visceral disease only (OR = 2.73; 95% CI: 2.04–3.64)), and a longer MFI time compared with de novo ABC (e.g., MFI 3–24 months (OR = 1.60; 95% CI: 1.22–2.10) and MFI ≥ 60 months (OR = 4.05; 95% CI: 3.18–5.15)). Furthermore, the HR + /HER2 + primary tumor subtype showed a reduced biopsy rate, as compared with the HR + /HER2- primary tumor subtype (OR = 0.68; 95% CI: 0.51–0.90).

### Subtype discordance rates

**Fig. 2:**
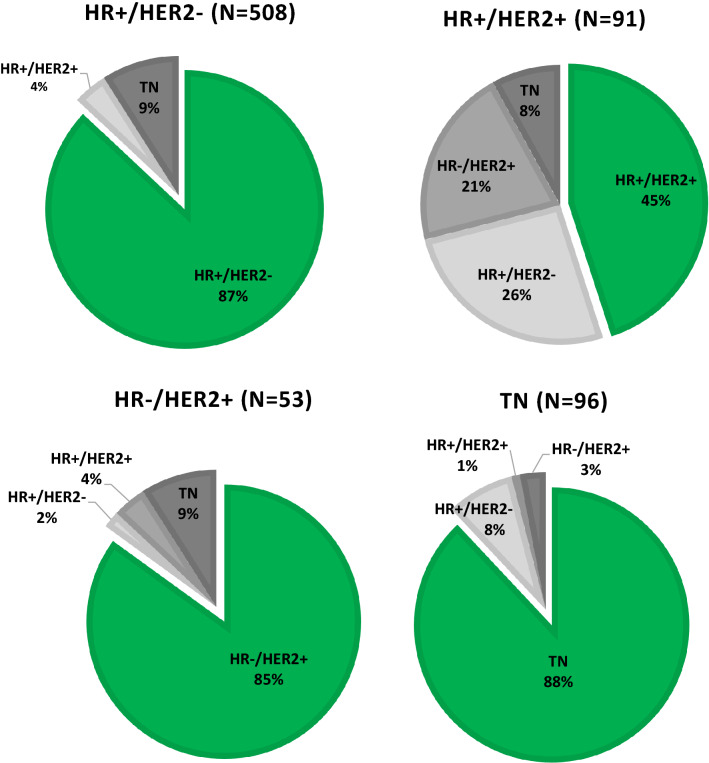
Subtype of the metastatic lesion per primary tumor subtype (*n* = 748)

**Fig. 3:**
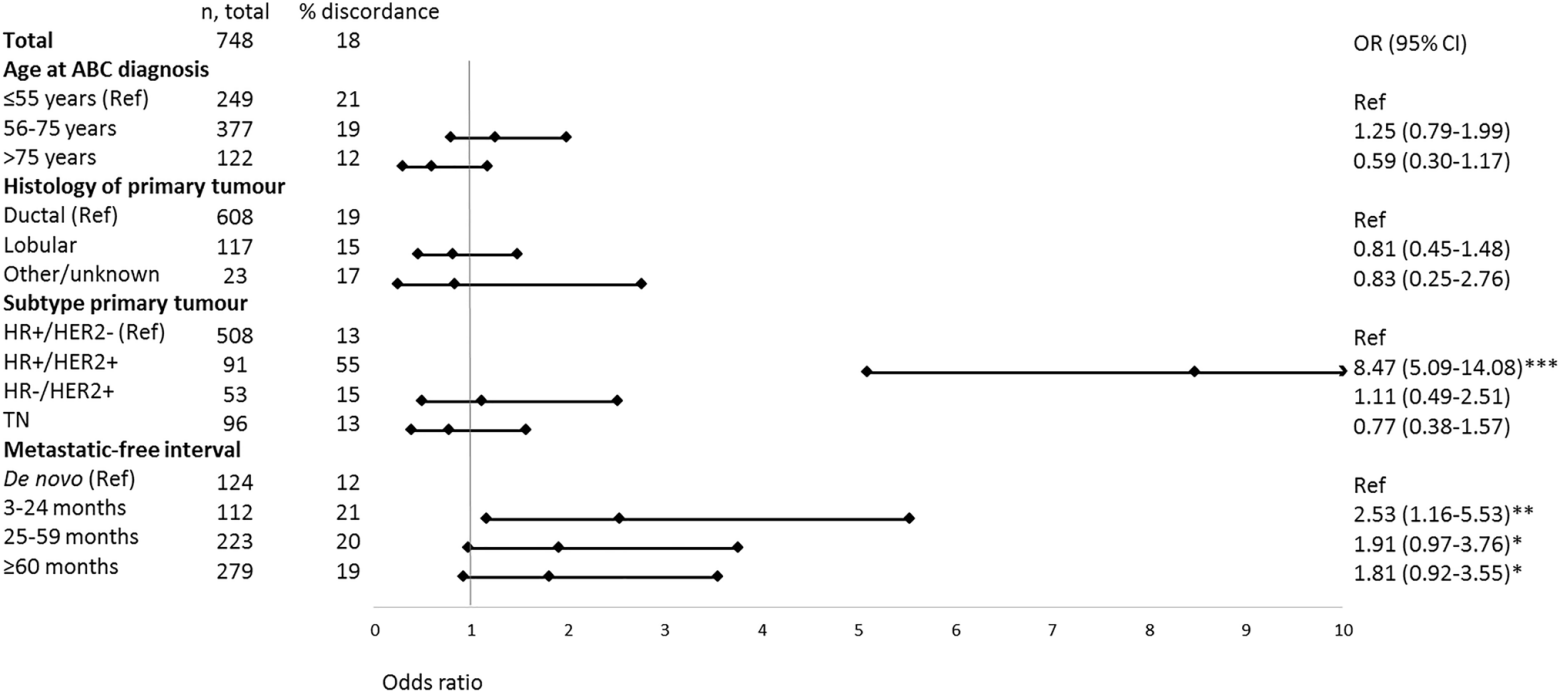
Factors associated with subtype discordance between the primary tumor and the metastatic site at time of ABC diagnosis, by multivariable logistic regression analysis. *Not statistically significant, but borderline (*P*< 0.10), ** *P*< 0.05, *** *P*< 0.001

After excluding 1712 patients with an incomplete receptor status of the metastasis, 362 patients with an incomplete receptor status of the primary tumor and 32 patients with two different primary tumor subtypes, 748 patients were eligible to study concordance and discordance rates between the metastasis and primary tumor (Table 1, Supplementary Figure S1). Among these patients, the overall receptor discordance rate was 18% (discordance rates for the individual receptors are presented in Supplementary Table S1). Discordance rates were 13% for HR + /HER2-, 15% for HR-/HER2 +, 12% for TN disease, and a high rate of 55% for HR + /HER2 + (Fig. 2, Supplementary Table S2). Patients with HR + /HER2 + disease converted mainly to HR + /HER2- disease (26%), followed by HR-/HER2 + disease (21%) and less often to TN disease (8%). Patients with HR + /HER2- and HR-/HER2 + disease converted mainly to TN disease (both 9%) and patients with TN disease converted mainly to HR + /HER2- disease (8%). The main predictor for discordance of receptor subtype between primary tumor and metastatic site was the HR + /HER2 + subtype of the primary tumor (versus HR + /HER2-: OR = 8.47; 95% CI: 5.09–14.08), followed by a MFI of 3–24 months (versus de novo*:* OR = 2.53; 95% CI: 1.16–5.53) (Fig. 3).

In the 624 patients diagnosed with metachronous metastases (excluding de novo ABC), prior HER2-targeted therapy in the adjuvant setting was associated with a threefold higher risk of discordance (versus no HER2-targeted: OR = 3.60; 95% CI: 2.05–6.35) (Table 2). However, after correcting for subtype and MFI, prior systemic treatment was no longer significantly associated with a higher incidence of discordance. The HR + /HER2 + subtype remained the only predictor significantly related to a higher discordance rate (OR = 7.49, 95% CI: 3.69–15.20). In addition, within the HR + /HER2 + subtype prior systemic treatment was not associated with a higher discordance rate (data not further shown).

**Table 2:** The impact of (neo-)adjuvant therapy on subtype discordance among patients with recurrent ABC (n = 624) (de novo metastatic disease excluded), multivariable analyses without and with adjustment for receptor subtype, and metastatic-free interval (MFI)

Predictors	Number of patients	Number of discordance	Multivariable analyses	
			Adjuvant therapies	Adjuvant therapies adjusted for subtype and MFI
	*N*	*N* (%)	OR (95% CI)	OR (95% CI)
Adjuvant endocrine therapy				
No	246	40 (16)	Ref	Ref
Yes	378	83 (22)	1.46 (0.94–2.27)*	0.97 (0.51–1.84)
Adjuvant HER2-targeted therapy				
No	555	93 (17)	Ref	Ref
Yes	69	30 (44)	3.61 (2.05–6.35)***	0.88 (0.37–2.09)
Adjuvant chemotherapy alone				
No	249	36 (15)	Ref	Ref
Yes	375	87 (23)	1.22 (0.76–1.96)	1.73 (0.99–3.01)*
Subtype primary tumor			–	
HR + /HER2-	423	62 (15)		Ref
HR + /HER2 +	76	43 (57)		7.49 (3.69–15.20)***
HR-/HER2 +	41	7 (17)		1.10 (0.35–3.44)
TN	84	11 (13)		0.65 (0.26–1.67)
MFI			–	
3–24 months	112	23 (21)		Ref
25–59 months	233	47 (20)		0.76 (0.41–1.42)
≥ 60 months	279	53 (19)		0.70 (0.38–1.32)

*ABC* advanced breast cancer, *HER2* Human Epidermal growth factor Receptor 2, *HR* hormone receptor, *MFI* metastatic-free interval, *OR* odds ratio

^*^Not statistically significant, but borderline (*P* < 0.10), ** *P* < 0.05, *** *P* < 0.001

### First-line treatment choices

**Fig. 4:**
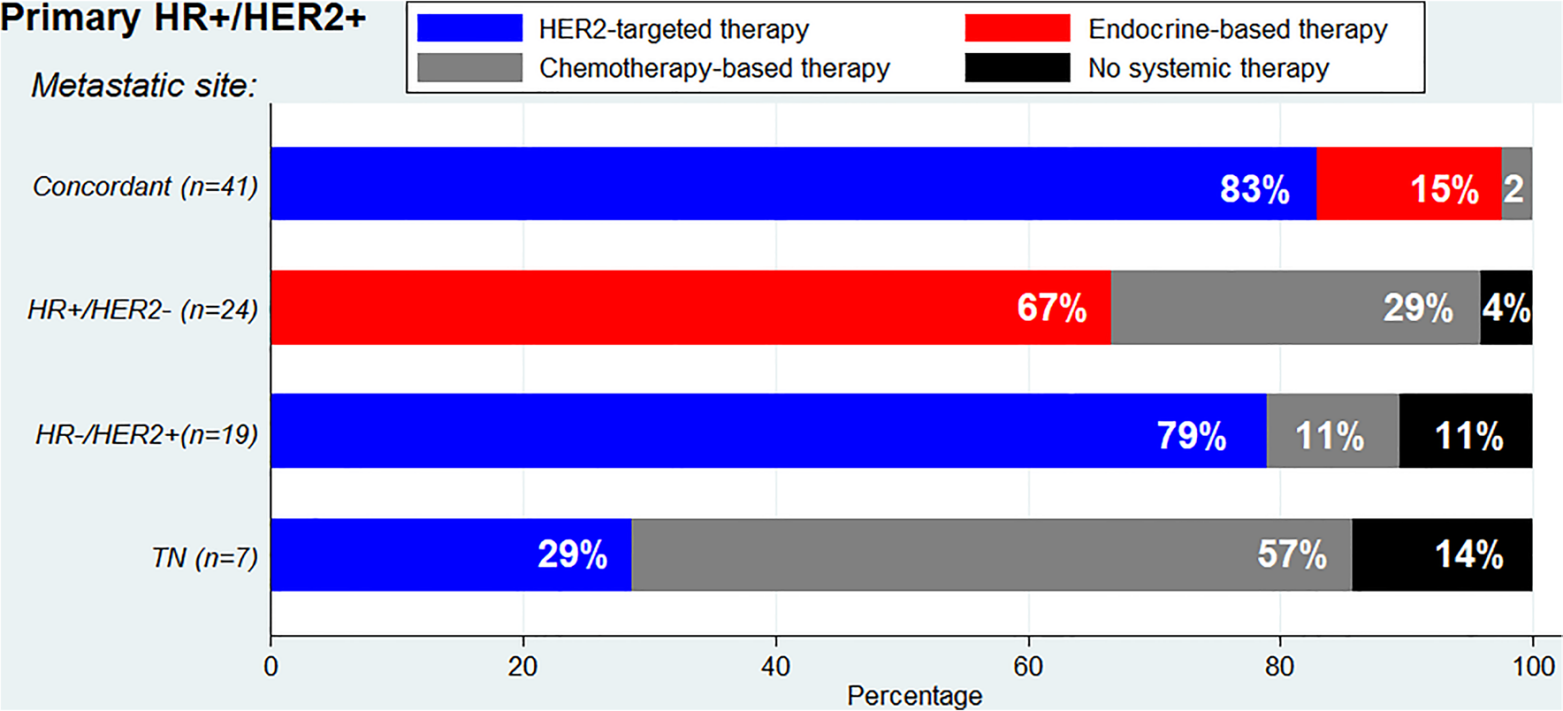
First treatment choice for concordant and discordant subtype of metastatic site in primary HR + /HER2 + subtype

First-line palliative systemic treatment choices were evaluated for the 91 patients with the HR + /HER2 + primary tumor subtype, where discordance was most prevalent. Of 41 patients with a concordant HR + /HER2 + subtype, the majority started with HER2-targeted therapy (83%), followed by first-line endocrine therapy (15%) (Fig. 4). Of 24 patients with receptor subtype changed into the HR + /HER2- subtype, 67% received first-line endocrine therapy and 29% chemotherapy. Of 19 patients with receptor subtype changed into the HR-/HER2 + subtype, 79% received HER2-targeted therapy and 11% chemotherapy. Of 7 patients with receptor subtype changed into the TN subtype, 57% received chemotherapy and 29% HER2-targeted therapy.

## Discussion

This study presents real-world data from the SONABRE registry on the biopsy rate of metastatic disease and receptor subtype discordance rate in 2854 patients diagnosed with ABC in 2007–2018. We demonstrated an overall biopsy rate of 60% and an overall subtype discordance rate of 18%. Surprisingly, the discordance rate in the HR + /HER2 + subtype was 55%. The HR + /HER2 + subtype itself was an independent predictor for subtype discordance, with no relation with prior adjuvant systemic therapies.

A biopsy at time of ABC diagnosis is recommended to confirm the diagnosis of breast cancer and to determine if subtype conversion has occurred [12]. We found a biopsy rate of 65% in all patients developing metastatic disease since 2013. It is a positive finding that the biopsy rate in our study increased over time, possibly as a result of the 2012 updated guideline, including the advice to biopsy a metastasis to confirm and characterize metastatic disease. Apart from the effects of the national breast cancer guideline revision itself, other factors may have contributed to the increasing biopsy rate. The hospitals included in the study are nearly all part of a regional cancer collaboration, OncoZON (Oncologic network Southeast Netherlands), and new guidelines are discussed in the OncoZON meetings. Next to this, enrollment in clinical studies more and more requires the obtainment of metastatic tissue, which might have increased the eagerness and rationale of a standard biopsy. More biopsies of a metastasis were obtained in patients with a longer metastatic-free interval, probably because the physician wanted to be certain of breast cancer recurrence. However, similar to prior studies, our results indicate that patients with a MFI of 3–24 months have an even high to higher risk of discordance, suggesting that conversion occurs more in fast-growing tumors [21, 24, 26]. This finding emphasizes the importance of increasing the use of biopsy assessments in daily clinical practice. In line with this recommendation, Criscitiello et al. have reviewed the pros and cons of metastatic biopsies, concluding that biopsies are needed to evaluate the molecular profile in patients to make an appropriate treatment plan [27].

In general, discordance between two biopsies of breast cancer disease could be explained by a measurement error. The possibility of a measurement error caused by limited diagnostic accuracy accounts for 5%–25% of discordance [28–30]. However, measurement errors alone do not explain the higher prevalence of the loss of a positive receptor rather than finding a new receptor positivity. Alternatively, breast cancer is known to be a heterogenic disease, meaning that different cell lines occur in terms of genetic and phenotypic features [31]. It is theorized that heterogeneity in one tumor consists of two different types: spatial heterogeneity and temporal heterogeneity [32]. Spatial heterogeneity refers to different cancer clones that vary in histologic or cellular morphology in different positions within a tumor localization or between metastases [31, 33]. Temporal heterogeneity describes the change of a tumor over time as an evolutionary process of tumor composition due to specific influencing factors as stress, tumor biological drift, and given therapies [27, 34]. Our study shows that subtype discordance was most prevalent in the HR + /HER2 + subtype, while earlier given (neo-)adjuvant systemic therapies were not related to discordance in the multivariate analysis. This result weakens the general assumption that therapeutic pressure is thought to have the biggest influence on receptor loss. We hypothesize that cross-talk between the ER and HER2 receptor stimulates discordance. It is assumed that ER expression leads to inhibition of the PI3K pathway and subsequently decreases the HER2 signal activity [35, 36]. Vice versa, amplified HER2 signal activity leads to a down-regulation of ER expression [37]. Nevertheless, the specific explanation for different subtypes within a disease remains uncertain. Repeated biopsies might be needed in case of irresponsive disease.

Nowadays, for daily clinical practice, the importance of receptor loss lies in preventing exposure to ineffective, potential toxic, and costly therapies and depriving the opportunity for appropriate therapies. In a previous prospective study by Amir et al. physicians changed treatment from the proposed plan in 14% of all biopsied patients [38]. This percentage also included change in treatment due to a benign lesion or malignancy from another primary origin. Of the 121 patients in the study, 41 (34%) had discordance in at least one receptor and of them 13 (32%) patients had a change in therapy. Our study design is not suitable to express the exact proportion of treatment change, since first-line treatment choices were not prospectively evaluated. However, based on the observed treatment patterns in concordant and discordant patients, we estimate that 40%–60% of all patients with subtype discordance would be treated differently, which is 5%–7% in all patients without a biopsy and even 30% in patients with the HR + /HER2 + subtype (Fig. 2, Supplementary table 3). We observed that for patients with the primary HR + /HER2 + subtype, the loss of HR positivity resulted in a total decline of endocrine therapy. For the loss of HER2 positivity, 29% of patients with a change toward a TN subtype according to metastatic biopsy continued to receive HER2-targeted therapy. The prescription of HER2-targeted therapy in these patients is not inherently wrong, considering spatial heterogeneity [39, 40]. Nevertheless, it is important to examine the current metastatic receptor subtype to be aware of discordance and the possible ineffectiveness of therapies targeted to the primary subtype.

The large population of patients diagnosed with ABC is a strength of this study. Our observational registry allows to evaluate physicians’ choices on obtaining a biopsy of metastatic site and given systemic therapies. Patient numbers were sufficient to perform multivariable analyses to find associated predictors. Furthermore, we only focused on patients diagnosed with ABC, so that the effect of MFI and (neo-)adjuvant therapies could be included in the logistic regression analyses. Our study also has certain limitations. The biopsy site was not registered, so for patients with multiple metastatic sites, we could not distinguish the actual biopsy site. It was also not registered whether biopsies were histological samples or possibly cytological, as both types of biopsies were included in the registry. However, we expect that most of the used biopsies were histological samples, as we only included biopsies where both HR and HER2 status were completely evaluated. Reproducibility of receptor evaluation in cytological samples has been a point of discussion, but seems to be reliable, depending on the technique used [41, 42]. Another confounder in the reproducibility of HER2 receptor status is the change in HER2 testing as recommended by ASCO, introduced in a guideline in 2007, and updated in 2013 and 2018 [43–45]. Since 2007 this led to reduced false-positive HER2 tests, while the updates led to a small increase of true positive HER2 tests [46]. Although this might have influenced the discordance rates in our study for a small proportion of patients, it does not explain the higher discordance rates in the HR + /HER2 + subtype in comparison to the other subtypes. Another limitation is that patient numbers were too low to perform a multivariable model per type of individual receptor, while our hypothesis is that these entities might have different predictors in the occurrence of subtype discordance. Nonetheless, our study provides realistic insight in biopsy rate and subtype discordance. We expect that the subgroup for evaluating the discordance rates is representative for the total population, as the distribution of primary subtype and individual receptor discordance was comparable with the total group (Table 1 and supplementary table S1). For future research, it might be interesting to evaluate the receptor subtype over repeated biopsies during ABC treatment, while therapeutic resistance occurs. Additionally, we emphasize the need for more research into biogenetic pathways and genetic mutations, as, in contrast to our expectations, the influence of adjuvant systemic therapies was not the reason for discordance.

## Conclusion

When deciding to start systemic therapy, it is important to perform a biopsy of a metastatic site to make sure that the chosen therapy targets all active receptors. Taking a biopsy is especially important in patients diagnosed with the HR + /HER2 + subtype of the primary tumor, where the majority of patients experienced discordance and which may be caused by a biological cross-talk between ER and HER2.

## Supplementary Information

Below is the link to the electronic supplementary material.

Supplementary file1 (pdf 184 KB)

## Data Availability

The data underlying this article will be shared on reasonable request to the corresponding author.
