# Supplementary material for: The initial hormone receptor/HER2 subtype is the main determinator of subtype discordance in advanced breast cancer: a study of the SONABRE registry
Source: Breast Cancer Res Treat. 2022 Jan 13;192(2):331–42. doi: 10.1007/s10549-021-06472-5 (PMC8926963; doi:10.1007/s10549-021-06472-5)
Supplement: Supplementary file 1 — Supplementary file1 (pdf 184 KB) [file 10549_2021_6472_MOESM1_ESM.pdf]

Supplementary Figure S1. Patient flow chart

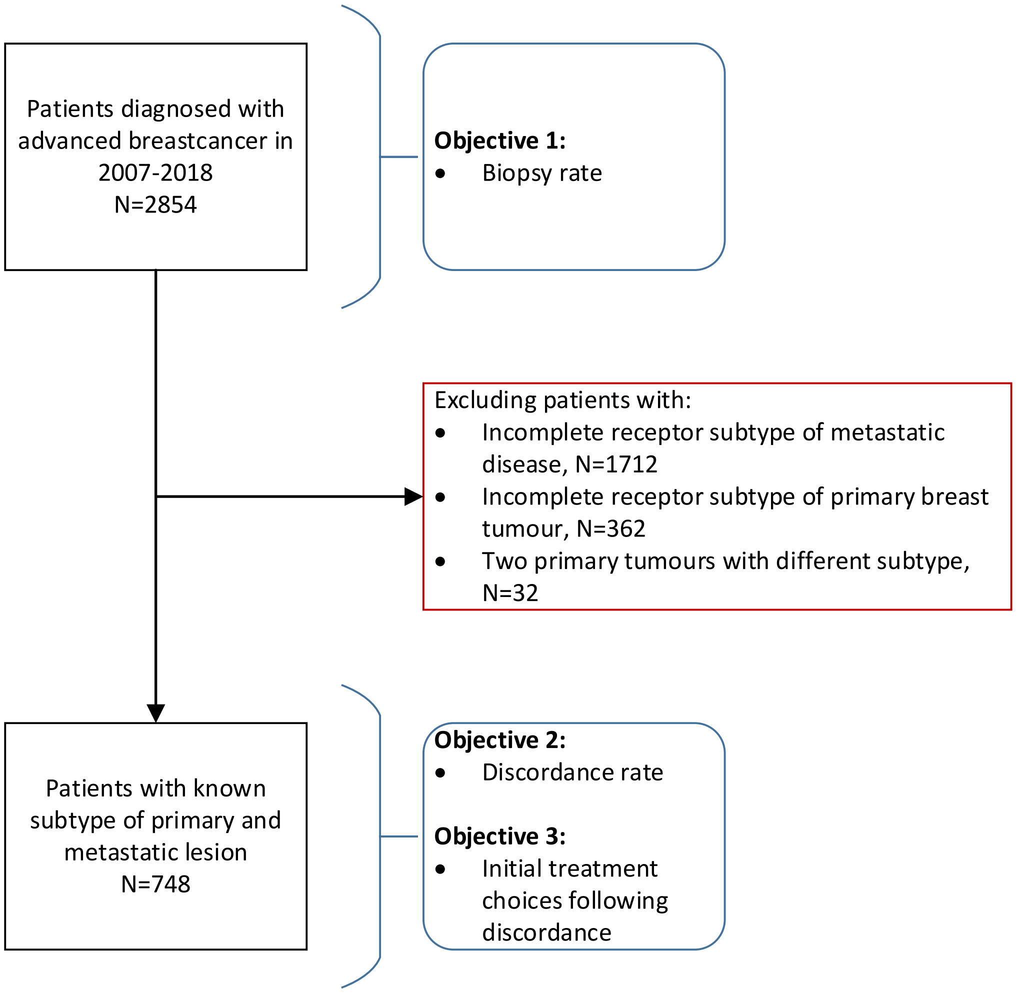

Supplementary Table S1. Change in estrogen receptor (ER), progesterone receptor (PR) and HER2 status between the primary breast cancer and a metastatic site, categorized by SONABRE total group (N=2854) and patients with known subtype of the primary and the mestastic site (N=748)

	**SONABRE Total group** (*N*=2854)		**SONABRE Known subtype of primary and metastatic site** (*N*=748)	
**Receptor status primary tumour**	*N*	*Discordance rate % (95% CI)*	*N*	*Discordance rate % (95% CI)*
ER total	1215	11% (10%-13%)	748	11% (9%-14%)
ER positive[a]	1011	11% (9%-13%)	593	12% (10%-15%)
ER negative	204	15% (11%-21%)	155	8% (4%-13%)
PR total	1136	34% (31%-37%)	744[b]	34% (30%-37%)
PR positive	746	45% (41%-48%)	447	47% (42%-51%)
PR negative	390	13% (10%-17%)	297	8% (5%-12%)
HER2 total	757	8% (7%-11%)	748	8% (7%-11%)
HER2 positive	610	25% (19%-33%)	144	26% (19%-33%)
HER2 negative	147	4% (3%-6%)	604	4% (3%-6%)

ER, Estrogen receptor; PR, progesterone receptor; HER2, Human Epidermal growth factor Receptor
[a]10% threshold for ER and PR positivity
[b]4 patients with missing PR-status

Supplementary Table S2. Subtype concordance and discordance rates between the primary and advanced breast cancer subtype

**Primary tumour subtype**	**Number of patients**	**Advanced breast cancer subtype**				**Discordance rate**
		HR+/HER2-	HR+/HER2+	HR-/HER2+	TN	
	N	*N (%)*	*N (%)*	*N (%)*	*N (%)*	%
**Total**	748					
HR+/HER2-	508	440 (87)	20 (4)	2 (0.4)	46 (9)	13%
HR+/HER2+	91	24 (26)	41 (45)	19 (21)	7 (8)	55%
HR-/HER2+	53	1 (2)	2 (4)	45 (85)	5 (9)	15%
TN	96	8 (8)	1 (1)	3 (3)	84 (88)	12%
**Recurrent disease**	624					
HR+/HER2-	423	361 (85)	16 (4)	2 (1)	44 (10)	15%
HR+/HER2+	76	20 (26)	33 (43)	16 (21)	7 (9)	57%
HR-/HER2+	41	1 (2)	1 (2)	34 (83)	5 (12)	17%
TN	84	7 (8)	1 (1)	3 (4)	73 (87)	13%

HER2, Human Epidermal growth factor Receptor 2; HR, hormone receptor; TN, triple negative

Supplementary Table S3. First-line treatment choice for concordant and discordant subtypes per primary tumour subtype

			**First-line treatment choice**			
**Primary tumour subtype**	**Subtype of metastatic site**	**Number of patients**	Endocrine-based therapy n (%)	HER2-targeted therapy n (%)	Chemotherapy-based therapy n (%)	No systemic therapy n (%)
HR+/HER2-	Concordant	440	345 (78)	0	82 (19)	13 (3)
	HR+/HER2+	20	8 (40)	7 (35)	4 (20)	1 (5)
	HR-/HER2+	2	0	1 (50)	1 (50)	0
	TN	46	7 (15)	0	36 (78)	3 (7)
HR+/HER2+	Concordant	41	6 (15)	34 (83)	1 (2)	0
	HR+/HER2-	24	16 (67)	0	7 (29)	1 (4)
	HR-/HER2+	19	0	15 (79)	2 (11)	2 (11)
	TN	7	0	2 (29)	4 (57)	1 (14)
HR-/HER2+	Concordant	45	1 (2)	30 (67)	3 (7)	11 (24)
	HR+/HER2-	1	0	0	0	1 (100)
	HR+/HER2+	2	0	2 (100)	0	0
	TN	5	0	0	4 (80)	1 (20)
TN	Concordant	84	0	2 (2)	67 (80)	15 (18)
	HR+/HER2-	8	2 (25)	0	6 (75)	0
	HR+/HER2+	1	0	1 (100)	0	0
	HR-/HER2+	3	0	2 (67)	0	1 (33)

HER2, Human Epidermal growth factor Receptor 2; HR, hormone receptor; TN, triple negative
